# The relationship between epilepsy and sexual dysfunction: a review of the literature

**DOI:** 10.1186/s40064-016-3753-5

**Published:** 2016-12-02

**Authors:** Muhammad Atif, Muhammad Rehan Sarwar, Shane Scahill

**Affiliations:** 1 Department of Pharmacy, The Islamia University of Bahawalpur, Bahawalpur, Punjab Pakistan; 2 School of Management, Massey University, Auckland, New Zealand

**Keywords:** Epilepsy, Sexual dysfunction, Antiepileptic drugs, Epidemiology, Relationship

## Abstract

**Background and objectives:**

Regardless of the disease states that people suffer from, maintaining sexual function is an important indicator of quality of life. The objective of this review was to figure out the relationship between epilepsy, antiepileptic drugs (AEDs) and sexual dysfunction.

**Results:**

In various epidemiological and clinical studies, epilepsy has been correlated with a reduction in sexual function. This sexual dysfunction is not always detected in epileptic patients until systematic efforts are put in place, as part of the assessment and treatment process. Therefore, precise evaluations of the incidence of treatment related sexual dysfunction in epileptic patients is still lacking.

**Conclusions:**

This literature review concluded that sexual function is influenced by the pathophysiology of epilepsy, as well as through the use of AEDs. To maximize quality of care in patients with epilepsy and those patients with other disease states who receive AEDs, it is important to address the status of the patient’s sexual function as part of the initial routine assessment and with any treatment related follow-up. Minimizing the effects of AED related sexual dysfunction can be achieved by raising awareness among patients, providing education and training for physicians regarding sexual dysfunction and obtaining a baseline sexual history from the patient so are important recommendations. In addition, systematic studies are needed to explore the risk and mechanism of such treatment related side effects on sexual function.

## Background

Historically discussion around sexual practices and associated dysfunction was taboo. However, in the modern era, awareness of issues associated with sexual dysfunction has been more commonly aired. Western civilization has become more open-minded about discussing sexual dysfunction, and in particular about erectile dysfunction (ED) in men, often associated with chronic disease. With this opening up of society there has been encouragement for people to think about their sexual performance and to seek advice from health consultants (Laumann et al. 1999; Kaufman et al. 2015).

The very first orally available drug for male ED was sildenafil (Viagra) which not only treated the problem but also through marketing campaigns, increased awareness among people. These days ED is considered amongst the general public to be a “medical condition” that can be managed. In terms of etiology both physical (ED in men due to circulatory disorders) and psychological factors (inadequate interpersonal associations, psychiatric illness) are responsible for sexual dysfunction. Another cause of sexual dysfunction is the use of drugs such as anti-hypertensive (Fogari et al. 1998), anti-psychotics (Baldwin and Birtwistle 1997), anti-depressants (Baldwin et al. 1997; Goldstein and Goodnick 1998) and anti-epileptic drugs (AEDs) (Hamed et al. 2015; Kirmani et al. 2014; Lombardi et al. 2015; Meryn 2015; Svalheim et al. 2015; Urso et al. 2014; Kaufman et al. 2015; Sivaraaman and Mintzer 2011; Kaufman and Struck 2011b). The link between sexual dysfunction and drug induced as opposed to disease induced effects is complicated, and the case of epilepsy and AEDs has received less attention than one might expect. Sexual dysfunction may impact on compliance and is reported to be dose dependent with various AEDs (Kaufman and Struck 2011a). For example, case reports of gabapentin-induced sexual dysfunction suggest that the minimum total daily dose required for sexual dysfunction is 900 mg (Dalal and Zhou 2008; GRANT and OH 2002).

## The human sexual response and sexual dysfunction

Natural sexual response of a human can be divided into four stages and disturbance can occur in any one these stages;

1. *Desire* typically this consists of fantasies about, and the desire to have, sexual activity.
2. *Excitement* the individual sense of sexual enjoyment, associated with physiological alterations, including penile tumescence and erection in men, and pelvic vasocongestion, inflamed external genitalia, and lubrication and extension of the vaginal canal in women.
3. *Orgasm* height of sexual enjoyment, by the discharge of sexual tension and rhythmic contraction of perineal muscles as well as reproductive organs.
4. *Resolution* sense of comfort and muscular leisure. Physiologically men are noncompliant to erection and orgasm for a varied time period, while women might be capable to respond to additional stimulation.

The label ‘sexual dysfunction’ has been classified in the International Classification of Mental and Behavioral Disorders, 10th edition (ICD-10) (World Health Organization 1992) (for those people who were incapable of enjoying their sexual relationship as they want). The ICD-10 sexual dysfunction categorization is not based on organic pathophysiology or infection. Both ICD-10 and Diagnostic and Statistical Manual of Mental Disorders, fourth edition (DSM-IV) (American Psychiatric Association 2000) utilized the same classificatory proposal (Table 1).

**Table 1:** Standard classification system for sexual dysfunction

F52.0	Lack or loss of sexual desire
F52.1	Sexual aversion and lack of sexual enjoyment
F52.2	Failure of genital response
F52.3	Orgasmic dysfunction
F52.4	Premature ejaculation
F52.5	Non-organic vaginismus
F52.6	Non-organic dyspareunia
F52.7	Excessive sexual drive
F52.8	Other sexual dysfunction, not caused by organic disorder or disease
F52.9	Unspecified sexual dysfunction, not caused by organic disorder or disease

Men and women can both suffer from sexual dysfunction the difference being women tend to express a lack of sexual enjoyment and/or interest, whilst men face sexual dysfunction related largely to physical response including failure to achieve an erection and/or premature ejaculation (McCabe et al. 2016). There has been dialogue to suggest that the categorical approaches to sexual dysfunction as defined by the ICD-10 and DMS-IV highlight the obscure, diverse and exclusive approaches whereby any person or couple could share their sexual problems (Bancroft 2009). It is evident if any phase of sexual response is changed, other phases may also be affected. Therefore, for diagnosis and subsequent treatment to be successful with sexual dysfunction it is essential to intervene with any relevant presenting complaint, rather than merely focusing on the standard diagnostic criteria.

## Epidemiology of sexual dysfunction

The epidemiology of sexual dysfunction has not been broadly discussed in the literature; or at least the literature has not been reviewed and synthesized. Thirty years ago prevalence data for sexual dysfunction were derived according to the DSM-III by Nathan et al., who analyzed twenty two studies about sexual dysfunction in the general population. Methodological issues in this analysis indicated that an extensive estimation might be made (Nathan 1986). The incidence of reduction of sexual desire was found to be 35% in women, 16% in men, premature ejaculation 35% and ED 10–20% in men and in female, orgasmic complication was found to be 5–15%. According to a study conducted in the United States (US), sexual dysfunction was more common in women (43%) than men (31%) (Laumann et al. 1999). In the case of women, low sexual desire (22% prevalence), arousal difficulty (14%) and sexual pain (7%) have been observed. Whilst for men premature ejaculation (21%), low sexual desire (5%) and ED (5%) have been observed. Sexual dysfunction rates can be different depending upon the population being studied and the nature of sexual dysfunction being evaluated. The data represented here demonstrates a high prevalence of sexual dysfunction for both males and females (Christensen et al. 2011; Ernst et al. 1993; Lindau et al. 2007; Hendrickx et al. 2016). Table 2 summarizes the key findings of studies published on sexual dysfunction among the general population.

**Table 2:** Studies of sexual dysfunction in the general population

Population	Subjects (M/F)	Dysfunctions assessed (%)	References
40-year-old Danish women	0/324	Impaired interest (41.8), orgasmic difficulties (36.7), dissatisfaction (35.4), pain/dyspareunia (1.3)	Garde and Lunde (1980)
35–59-years-old Oxford women	0/521	Impaired interest (17), orgasmic difficulties (16), pain/dyspareunia (8)	Osborn et al. (1988)
29-year-old men, 30-year-old women	197/218	*Men* dissatisfaction (21), impaired interest (7), premature ejaculation (4)	Ernst et al. (1993)
		*Women* dissatisfaction (21), impaired interest (16), orgasmic difficulties (7), pain/dyspareunia (6)	
Iranian women aged 20–60-years-old	0/2626	Orgasmic difficulties (37), low sexual desire (35), lubrication disorders (33.7), dissatisfaction (31.5), arousal disorders (30), pain/dyspareunia (26.7)	Safarinejad (2006)
US adults (57–85-years of age)	1455/1550	*Men* erectile difficulties (37), lack of interest in sex (28), climaxing too quickly (28), anxiety about performance (27), inability to climax (20)	Lindau et al. (2007)
		*Women* lack of interest in sex (43), lubrication disorders (39), inability to climax (34), finding sex not pleasurable (23), pain (17)	
British women aged 18–85-years-old.	0/1489	Low sexual desire (17.3), dissatisfaction (14.6), orgasmic difficulties (13.5), arousal disorders (13.3), lubrication disorders (11.5), pain/dyspareunia (11.5)	Burri and Spector (2011)
Danes aged 16–95-years-old	2120/2295	*Men* premature ejaculation (7), erectile dysfunction (5), anorgasmia (2), dyspareunia (0.1)	Christensen et al. (2011)
		*Women* lubrication insufficiency (7), anorgasmia (6), dyspareunia (3), vaginismus (0.4)	
Swiss men aged 18–25-years-old	3886/0	Erectile dysfunction (29.9), premature ejaculation (11.4)	Mialon et al. (2012)
Flemish men and women 14–80-years-old	651/695	*Men* erectile dysfunction (7.1), premature ejaculation (6.3), hyperactive sexual desire (3.3)	Hendrickx et al. (2016)
		*Women* lubrication dysfunction (9.6), absent or delayed orgasm (7.4), low responsive sexual desire (6.9), low spontaneous desire (5.3)	

The major categories of sexual dysfunction are influenced by various aspects. First, the means of enquiry and type of data reporting mechanism i.e. the occurrence of sexual dysfunction in one prospective study was 14% when relying on spontaneous self-reporting, but 58% when the doctor directly asked the patient about it (Montejo-Gonzalez et al. 1997). Second, the attitudes of people with respect to disclosing and fully discussing their sexual dysfunction issues with their physician is strongly influenced by the culture of a society (Bhavsar and Bhugra 2013).

To provide an example, this is especially the case with Muslim people whereby discussing anything sexually-related, including issues like sexual dysfunction outside of marriage is prohibited. This can make it very difficult for Muslim clients to seek help from health professionals; however, there mechanisms in place where it may be allowed for treatment purposes (Sungur and Bez 2016). Any sexual activity outside of marriage is also banned under Islam, but the religion encourages the enjoyment of sex and sensitivity to the needs of the spouse within heterosexual marriages. Therefore, it is critical for health professionals to be aware of the role of culture in defining sexual dysfunction and how cultural factors can be considered when initiating treatment as well as in therapeutic engagement and alliance between patient and practitioner.

Third, sexual dysfunction can be defined in many terms and is reliant on the belief of what is usual or normal in a sexual relationship (Bhugra and de Silva 2007). Finally, temporal trends can occur with spikes in concern about disorders, as increased awareness of sexual matters and availability of medical treatments increase the numbers who perceive themselves as suffering from sexual dysfunction and come forward to seek treatment (Baldwin 2001).

## Sexual dysfunction in epileptic patients

Epilepsy is a significant chronic neurologic disorder characterized by episodes of seizure (of various types), which generally require lifelong management with medication (Atif et al. 2016). Children and adults are both affected by this disease. Approximately 1 in 26 people will develop epilepsy at some point in their lives (England et al. 2012). Sexual dysfunction associated with epilepsy is not yet completely understood (Harden 2008) and epilepsy is a complex disease causing several downstream pathological alterations which may need further exploration and treatment. One manifestation of the disease includes endocrine disorders in men and women which influences the reproductive system. Reproductive endocrine disorders in epileptic women include polycystic ovary syndrome (PCOS) (Bauer and Cooper-Mahkorn 2008; Bilo et al. 1988; Herzog et al. 2003a, b, 1984; Hopkinson et al. 1998; Gorkemli et al. 2008), hypothalamic amenorrhea (Bilo et al. 2001; Herzog et al. 1986b), hyperandrogenemia (HA) (Luef et al. 2002b; Löfgren et al. 2007), galactorrhea, hirsutism, menstrual abnormality and infertility (Bauer 2001; Bauer et al. 2002; Herzog et al. 2003b; Löfgren et al. 2006). Sexual dysfunction impacts between one and two thirds (30–66%) of epileptic men (Blumer and Walker 1967; Hierons and Saunders 1966; Herzog et al. 1986a) and between 14 and 50% of epileptic women (Demerdash et al. 1991; Jensen 1992). Evidence suggests that (Edwards et al. 1999; Baird et al. 2003; Quigg et al. 2002; Morrell et al. 2005) patients with partial epilepsy (PE) are more severely affected by reproductive endocrine and sexual dysfunction than idiopathic generalized epileptic (IGE) patients, predominantly of temporal lobe origin (Table 3).

**Table 3:** Studies of epilepsy type and sexual and reproductive dysfunction

Dysfunction assessed	Epilepsy type	Sex (n)	Prevalence	Sexual dysfunction assessment	References
*Sexual*					
i. Global anorgasmia ii. Vaginismus iii. Dyspareunia	PE	F (99)	i. 17.9% ii. 27.8% iii. 38.5%	Questionnaire	Morrell and Guldner (1996)
	IGE	F (17)	i. 31.3% ii. 13.3% iii. 18.8%		
i. Sexual arousability ii. Sexual anxiety	PE	F (99)	i. 72.2% ii. 15.4%	Questionnaire	Lambert (2001)
	IGE	F (17)	i. 84.2% ii. 7.6%		
Erectile dysfunction	GTCS	M (25)	72%	Questionnaire	Nikoobakht et al. (2007)
	PE	M (16)	32%		
Erectile dysfunction	PE	M (418)	25.8%	Clinical interview	Keller et al. (2012)
	IGE	M (166)	29.5%		
*Reproductive*					
i. Menstrual disorders ii. PCO iii. HA iv. PCOS	PE	F (90)	i. 44% ii. 46% iii. 33% iv. 44%	Clinical examination, transvaginal ultrasonography	Löfgren et al. (2007)
	IGE	F (57)	i. 29% ii. 28% iii. 13% iv. 19%		
i. Menstrual disorders ii. Hirsutism iii. PCO	IGE	F (52)	i. 23% ii. 5.8% iii. 25%	Clinical examination, transvaginal ultrasonography	Luef et al. (2002a)
	PE	F (40)	i. 42.5% ii. 2.5% iii. 37.5%		
PCO	IGE	F (36)	21%	Suprapubic ovary ultrasound	Murialdo et al. (1997)
	PE	F (65)	14.5%		
i. Hypomenorrhoea ii. Amenorrhea iii. Oligomenorrhoea iv. Polymenorrhoea	IGE	F (21)	Overall results i. 22% ii. 16% iii. 9% iv. 2%	Clinical examination	Demerdash et al. (1991)
	PE	F (106)			

*PCO* polycystic ovaries, *HA* hyperandrogenism, *PCOS* polycystic ovary syndrome, *IGE* idiopathic generalized epilepsy, *PE* partial epilepsy, *F* female

The underlying mechanism of reproductive dysfunction has various hypotheses. One of the most accepted hypotheses suggests that the activity of gonadotropin-releasing hormone (GnRH) pulse generator is disturbed due to the temporal lobe regions associated with epilepsy, which might be the origin of paroxysmal release, just before the hypothalamus (Verrotti et al. 2015). Amplification of the pulse frequency of GnRH and as a result the luteinizing hormone (LH) and follicle-stimulating hormone (FSH) ratio is related among PCOS patients (Rauchenzauner et al. 2014). The decrease in GnRH pulse frequency also lowers LH and estrogen (E2) levels, which an attribute is of HA. Moreover, several other aspects, such as, obesity, genetic disposition, laterality of epilepsy, type of AED prescribed, age and severity of seizures, might also be reasons for clinical expression of specific reproductive disorders i.e. HA, PCOS, ovulation, hirsutism (Verrotti et al. 2011).

## The relationship between sexual dysfunction and AEDs

Seizure disorders are linked with sexual dysfunction as part of the pathophysiology, whilst all the available AEDs have not yet been definitively proven to have negative impacts on sexual function. As such, evaluation of AED-induced sexual dysfunction is complicated and less than clear (Crenshaw and Goldberg 1996; Gitlin 2003). Comparative analyses of AEDs in patients with epilepsy, inducing sexual dysfunction are hard to find. The enzyme stimulating AEDs (phenytoin, topiramate, phenobarbital, oxcarbazepine and carbamazepine) raise hepatic synthesis of sex hormone binding globulin (SHBG) that lessens the accessibility of testosterone. Additionally, enhanced sex hormone metabolism and contraceptive hormones are not found with the use of AEDs that do not stimulate hepatic enzymes (e.g. lamotrigine, valproate, gabapentin, and vigabatrin) (Herzog et al. 2005; Morrell 2003). A consistent finding in the literature is that sexual dysfunction occurs with greater prevalence in patients taking phenytoin and carbamazepine than in patients taking valproate or lamotrigine (Herzog et al. 2005; Gutierrez et al. 2008). Women treated with valproate for bipolar disorder have also been reported to have severely decreased libido and anorgasmia, while there are case reports of gabapentin-associated anorgasmia in women who suffer from epilepsy (Harden 2005). A review of pregabalin efficacy and tolerability makes no mention of sexual dysfunction as an observed adverse effect (Tassone et al. 2007). Several studies in men and women have indicated that AEDs are related to sexual dysfunction (Table 4).

**Table 4:** Anti-epileptic drugs associated with sexual dysfunction

Drug and dose (mg)	Dysfunction assessed	Sex (n)	Prevalence	Diagnosis	Sexual dysfunction assessment	References
*Sexual dysfunctions*						
Primidone (250)	Decreased libido or impotence	M (109)	22%	GTCS	Questionnaire	Mattson et al. (1985)
Carbamazepine (580)	Sperm abnormality	M (13)	77%	IGE, PE	Semen analysis and ultrasonography of testicles	Isojärvi et al. (2004)
Oxcarbazepine (1060)		M (17)	59%			
Valproate (1080)		M (25)	80%			
Topiramate (100–200)	Erectile dysfunction	M (40)	5%	PE	Clinical interview	Holtkamp et al. (2005)
Valproate (400–1250)	i. Erectile dysfunction ii. Premature ejaculation iii. Orgasm dysfunction iv. Diminished sexual desire	M (8)	i. 50% ii. 50% iii. 0.0% iv. 62.5%	GTCS	Sexual function questionnaire	Hamed et al. (2006)
Carbamazepine (400–1000)		M (17)	i. 47.1% ii. 35.3% iii. 5.9% iv. 52.9%			
Pregabalin (150–600)	Erectile dysfunction	M (363)	3.0%	PE	Questionnaire	Hitiris et al. (2006)
Lamotrigine (50–200)	Lack of sexual interest	M (75), F (66)	11%	Complex PE, IGE	Changes in sexual functioning questionnaire (CSFQ)	Gil-Nagel et al. (2006)
Phenytoin (100)	Retrograde ejaculation	M (1)	Case report	PE	Clinical examination	Elia et al. (2010)
Zonisamide (200)	Erectile dysfunction	M (1)	Case report	PE	Clinical interview	Maschio et al. (2011)
Gabapentin (900)	Anorgasmia	F (1)	Case report	GTCS	Clinical interview	Perloff et al. (2011)
Leviteracetam (2000–3000)	i. Decreased libido ii. Erectile dysfunction iii. Anhedonia	M (2)	Case reports	GTCS, Myoclonic seizures	Clinical interview	Calabrò et al. (2012)
Pregabalin (300)	Anorgasmia	M (4)	Case reports	PE	Clinical interview	Calabrò et al. (2013)
Leviteracetam (500–1000)	Sperm abnormality	M (10)	69.2%	GTCS, complex PE	Computer-aided sperm analysis	Xiaotian et al. (2013)
Valproate (500–1000)		M (15)	18.8%			
Lacosamide (400)	i. Loss of libido ii. Erectile dysfunction	M (1)	Case report	Simple and complex seizures	Clinical examination	Calabro (2016)

*GTCS* generalized tonic–clonic seizure, *PE* partial epilepsy, *IGE* idiopathic generalized epilepsy

## Antiepileptic drugs, epilepsy and serum sex hormones

Sex hormones levels contribute to sexual dysfunction as well as other reproductive disorders and these levels can be change as a result of the administration of AEDs (Table 5) (Morrell et al. 2005). The reproductive endocrine system may also be influenced by the pathophysiological effects of epilepsy as a disease in its own right (Scharfman et al. 2008). Endocrinological alterations due to epilepsy can be postulated in spite of the complicated interconnection among the limbic system and the hypothalamic–pituitary axis (HPA). Production toward HPA from the limbic cortex, containing nuclear configuration inside the amygdale, can alter key features in the discharge of sex hormones. Reproductive functions and discharge of sex hormones are affected by the influence of epilepsy in the medial temporal lobe region of the brain (Herzog et al. 1986b). Though the complexity it remains uncertain as to whether hormonal irregularity is because of epilepsy associated HPA dysfunction or whether it is due to the side effects of AEDs (Verrotti et al. 2011). The role of the HPA, the production of LH and FSH, GnRH, prolactin (PRL), and the end products of metabolism and their concentrations i.e. E2, T (testosterone), and DHEAS (dehydroepiandrosterone), are altered in epileptic women (Table 5) (Morrell 2003; Genton et al. 2001).

**Table 5:** Serum sex hormone and sex hormone-binding globulin (SHBG) concentrations in various studies

AEDs	Sex (n)	SHBG	T	FT	DHEAS	DiT	FAI	E2	A	Comments	References
Carbamazepine, valproate	M (37)	↑	↓	↓	NA	NA	NA	NA	NA	Libido and fertility are low in epileptic patients	Dana-Haeri et al. (1982)
Carbamazepine, valproate, phenobarbital	M (72)	↑	↑	↓	↓	↑	↓	NA	NA	–	Toone et al. (1983)
Carbamazepine	M (06)	↑	↑	↓	↓	NA	NA	NA	NA	Induces changes in circulating androgen concentrations	Connell et al. (1984)
Phenytoin	M (20)	NA	↑	↓	NA	NA	NA	NA	NA	Reductions in free testosterone serum levels are associated with TLE in men	Herzog et al. (1986a)
Carbamazepine	F (23)	↑	≠	≠	↓	NA	↓	≠	NA	1 year prospective data	Isojärvi (1990)
Carbamazepine	M (21)	↑	≠	≠	NA	NA	↓	↓	NA	1 year prospective study	Isojärvi et al. (1991)
Carbamazepine	F (32)	↑	≠	NA	NA	NA	≠	↓	NA	Low estradiol/SHBG ratios associated with menstrual disorders	Isojarvi et al. (1995)
Carbamazepine, phenytoin, phenobarbital	M (37)	↑	≠	↓	↓	↓	NA	NA	NA	Decreased T/E2 ratio associated with hyposexuality	Murialdo et al. (1995)
carbamazepine	M (25)	↑	≠	NA	NA	NA	↓	NA	NA	High SHBG levels associated with diminished potency	Isojärvi et al. (1995)
Valproate, carbamazepine	F (65)	↓	↑	NA	↑	NA	NA	NA	≠	T concentration is more elevated in women initiating treatment at the age <20 years	Isojärvi et al. (1996)
Carbamazepine	F (23)	≠	↓	NA	↓	NA	≠	↓	NA	–	Murialdo et al. (1998)
Valproate	F (41)	≠	↑	NA	NA	NA	↑	≠	NA	Pre-pubertal, pubertal and Post-pubertal girls aged 8–18 year	Vainionpää et al. (1999)
Valproate	F (10)	↑	↑	NA	NA	NA	≠	≠	↑	3 month prospective study	Rättyä et al. (2001)
Carbamazepine, lamotrigine, valproate, oxcarbazepine	M (70)	≠	≠	NA	≠	↑	NA	NA	NA	AEDs induce changes in circulating androgen concentrations	Mikkonen et al. (2004)
Carbamazepine	F (25)	↑	↓	NA	≠	NA	↓	≠	≠	Low E2/SHBG ratio is not associated with menstrual disorders	Löfgren et al. (2006)
Valproate, leviteracetam	M/F (20)	≠	≠	NA	≠	NA	NA	≠	↑	–	Rauchenzauner et al. (2010)
Topiramate	M (36)	NA	↑	↓	↓	NA	NA	NA	↓	Hormonal changes are associated with sexual dysfunction	Urso et al. (2014)
Primidone	M/F (20)	↑	↓	NA	NA	NA	NA	↓	NA	Leads to infertility in epilepsy patients	Luef and Madersbacher (2015)
Lacosamide	M (10)	↓	↑	NA	NA	NA	↑	NA	NA	Cause deinduction of CYP enzymes	Elger et al. (2016)

*AEDs* anti-epileptic drugs, *M* male, *F* female, *T* testosterone, *DHEAS* dehydroepiandrosterone, *DiT* dihydrotestosterone, *FT* free testosterone, *FAI* free androgen index (100 × T/SHBG), *E2* estradiol, *A* androstenedione, *TLE* temporal lobe epilepsy, *NA* not available

**≠**, unchanged; ↑, increased; ↓, decreased

## Treatment of sexual dysfunction

Appropriate treatment to be provided for epileptic patients with sexual dysfunction requires evaluation according to patient requirements, epileptic condition and comorbidities and the medicines available for managing epilepsy. Various approaches might be helpful for treatment of sexual dysfunction in epileptic patients including;

- Behavioral approaches to enhance sexual performance.
- Waiting for tolerance to develop.
- Dose reduction of current medicines.
- Drug administration delayed until after sex.
- Adjuvant treatment of the sexual dysfunction per se.
- Varying the diverse range of antiepileptic’s available for individual patients.

Several adjuvant medicines i.e. buspirone, yohimbine, neostigmine, cyproheptadine, mianserin, amantadine and dexamphetamine, are used to cure AEDs related sexual dysfunction (Shapira 2003). ED in men can be treated by using indicative approaches in combination with previously described treatment approaches. This might include corporal initiation of the penis to stimulate spinal reflexes, and loss of blood can be prevented by inserting elastic constricting bands at the base, and negative pressure facilitating cavernous blood flow generated by using vacuum devices (Guldner and Morrell 1996). Additionally, methods such as the intercavernosal injection of vasodilators, i.e. papaverine or a grouping of papaverine, phentolamine, and prostaglandin E1, or yohimbine can be used to treat ED (Smaldone et al. 2004; Rokkas 2015). Other pharmaco-therapies for combatting ED to enhance the quality and frequency of erection include; topical nitroglycerine, topical minoxidil, and sildenafil (Rokkas 2015). The authors are not aware of any research that has been conducted on the use of sildenafil for ED in epileptic patients. Phosphodiesterase type-5 inhibitor drugs are now the first-line treatment for ED (Shamloul and Ghanem 2013; Bruzziches et al. 2013; Lee et al. 2015). Selective serotonin reuptake inhibitors (for example; escitalopram, paroxetine, fluoxetine, sertraline, etc.) and topical anesthetic creams are available but are not approved for the ED indication. However, they still serve as effective off-label treatments for premature ejaculation (Stimmel and Gutierrez 2006). Testosterone and aromatase inhibitors have been used in the investigational setting to treat sexual dysfunction in men taking AEDs (Hamed et al. 2013). Patient education and follow-up appointments are essential to ensure the optimal outcomes of pharmacologic treatment for sexual dysfunction in the epileptic patient. There are no approved pharmacologic treatments for female hypoactive sexual desire disorder or female orgasmic disorder. However, female sexual arousal disorder is treated with either estrogen replacement therapy or vaginal lubricants (Gutierrez et al. 2008).

## Conclusions and recommendations

Sexual dysfunction in the epileptic patient is complex as it is often difficult to determine whether the dysfunction is disease or treatment related. There is a scarcity of data on the occurrence of sexual dysfunction among the population as a whole and amongst those taking AEDs which can induce sexual dysfunction. This is a broad review looking at sexual dysfunction but the primary aim is to better understand the literature surrounding sexual dysfunction in the epileptic patient where both the pathophysiology and the treatment can cause a degree of sexual dysfunction. This review concluded that the most common sexual dysfunction in males is ED and early ejaculation while females complain of lack of interest in sex and failing to reach orgasm.

Epilepsy is associated with sexual dysfunction while it is not yet known whether all AEDs have a negative impact on sexual function. Unraveling the causations of AED-induced sexual dysfunction in the epileptic patient is complicated. Sex hormone levels can be changed due to AEDs which are the cause of sexual dysfunction and reproductive disorders. Systematic studies are needed to explore the risks and mechanisms of such treatment related side effects on sexual function; particularly in epileptics. This review lead to the following recommendations for physicians in improving the management of sexual dysfunction for their patients: (1) improved electronic medical record template to include appropriate questions related to sexual dysfunction in appropriate populations such as epileptics; (2) revision of undergraduate and residency curricula to include a broader appreciation of the impact of illness processes, comorbidities, and associated pharmacotherapies upon sexual functioning; and (3) required continuing medical education on sexual dysfunction.

## Abbreviations

AEDs: antiepileptic drugs
ED: erectile dysfunction
ICD-10: International Classification of Mental and Behavioral Disorders, 10th edition
DSM-IV: Diagnostic and Statistical Manual of Mental Disorders, fourth edition
DHEAS: dehydroepiandrosterone
DiT: dihydrotestosterone
FT: free testosterone
FAI: free androgen index
E2: estradiol
A: androstenedione
TLE: temporal lobe epilepsy
PE: partial epilepsy
IGE: idiopathic generalized epilepsy
